# Effects of forced movements on learning: Findings from a choice reaction time task in rats

**DOI:** 10.3758/s13420-016-0255-9

**Published:** 2017-01-14

**Authors:** Hidekazu Kaneko, Hiroto Sano, Yasuhisa Hasegawa, Hiroshi Tamura, Shinya S. Suzuki

**Affiliations:** 1 National Institute of Advanced Industrial Science and Technology (AIST), AIST Tsukuba Central 6, Higashi, Tsukuba, Ibaraki 305-8566 Japan; 2 University of Tsukuba, Tsukuba, Ibaraki Japan; 3 Osaka University, Suita, Osaka Japan

**Keywords:** Actuator, Behavior, Proprioceptive sensation, Voluntary movement, Robotics

## Abstract

To investigate how motor sensation facilitates learning, we used a sensory–motor association task to determine whether the sensation induced by forced movements contributes to performance improvements in rats. The rats were trained to respond to a tactile stimulus (an air puff) by releasing a lever pressed by the stimulated (compatible condition) or nonstimulated (incompatible condition) forepaw. When error rates fell below 15%, the compatibility condition was changed (reversal learning). An error trial was followed by a lever activation trial in which a lever on the correct or the incorrect response side was automatically elevated at a preset time of 120, 220, 320, or 420 ms after tactile stimulation. This lever activation induced forepaw movement similar to that in a voluntary lever release response, and also induced body movement that occasionally caused elevation of the other forepaw. The effects of lever activation may have produced a sensation similar to that of voluntary lever release by the forepaw on the nonactivated lever. We found that the performance improvement rate was increased by the lever activation procedure on the incorrect response side (i.e., with the nonactivated lever on the correct response side). Furthermore, the performance improvement rate changed depending on the timing of lever activation: Facilitative effects were largest with lever activation on the incorrect response side at 320 ms after tactile stimulation, whereas hindering effects were largest for lever activation on the correct response side at 220 ms after tactile stimulation. These findings suggest that forced movements, which provide tactile and proprioceptive stimulation, affect sensory–motor associative learning in a time-dependent manner.

*Motor sensation* is defined as sensation induced by body movements, including cutaneous and proprioceptive sensations induced by voluntary and involuntary movements. Motor sensation provides feedback that enables perception of the body’s position and accurate movements during motor learning (Brindle, Mizelle, Lebiedowska, Miller, & Stanhope, 2009; Khoshnoodi, Motiei-Langroudi, Omrani, Ghaderi-Pakdell, & Abbassian, 2006). Thus, a lack of motor sensation causes inaccurate movements. For example, if tactile sensation of the tongue is diminished by treatment with lidocaine or capsaicin, the accuracy with which the subject can control a prosthetic device with the tongue is reduced (S. A. Boudreau, Hennings, Svensson, Sessle, & Arendt-Nielsen, 2010). Motor sensation is also used in cognitive learning. Cutaneous and proprioceptive sensations induced by finger and arm movements can facilitate recognition of an object’s shape through touch (Heller, 1984; Heller & Myers, 1983; Yoshioka, Craig, Beck, & Hsiao, 2011). Thus, motor sensation is involved in various learning processes.

Induction and emphasis of motor sensation may facilitate learning. For example, in rehabilitation, walking speed and distance improve through forced movement (Colombo, Joerg, Schreier, & Dietz, 2000; Fleerkotte et al., 2014; Hesse, Uhlenbrock, Werner, & Bardeleben, 2000; Hidler et al., 2009; Hornby et al., 2008). In addition, emphasis of motor sensation by application of additional tactile signals facilitates cognitive training for detection of cancers by palpation (Gerling & Thomas, 2005). These findings indicate that performance improvement is facilitated by motor sensations or associated sensory information transmitted to the brain (M. J. Boudreau & Smith, 2001; Gioanni, 1987).

Because it provides continuous discriminative afferent signals critical to response execution (Notterman & Mintz, 1965), motor sensation is typically investigated in a paradigm of closed-loop-control processes, as in the reports cited above. However, we demonstrated that it also contributes to trial-by-trial learning of a sensory–motor association task that does not require closed-loop control in order to make a successful response (Sano, Kaneko, Hasegawa, Tamura, & Suzuki, 2013). As a result, we hypothesized that, if activities of cutaneous and proprioceptive afferents induced by forced movements are similar to those observed in voluntary correct-response movements, then such forced movements can facilitate task performance improvement. In the rehabilitation field, Ethier, Gallego, and Miller (2015) recently suggested that appropriate plasticity in the peripheral and central nervous system can be facilitated by associating motor intent with artificially generated movement and afferent activity. These findings indicate that motor sensation plays an important role even in learning tasks that do not require closed-loop control, and that suitable interaction of sensation with motor intent can facilitate learning.

In this study, we hypothesized that interaction of motor sensation with motor intent is necessary to facilitate learning in a choice reaction time task. If that were the case, motor sensation applied at the timing of motor intent would produce more effective performance improvement. Although motor intent is difficult to detect in practice (Chase, Schwartz, & Kass, 2010; Gabbard, 2009), it must arise before and continue during voluntary response movements. Therefore, we should be able to facilitate performance improvement by applying motor sensation temporally close to response movements.

To determine whether such timing effects exist, we investigated how the performance improvement rate was modulated by motor sensation at different timings after the task cue stimulus of a choice reaction time task in rats. This rate was evaluated using reaction time (RT) and error rate (ER). If rats could perform the task with shorter RT and lower ER in fewer training days, performance improvement was considered to be faster. In the choice RT task we used, rats were trained to press a lever with each forepaw and respond to a tactile stimulus (air-puff) applied to the right or left forepaw by releasing one (correct response side) of the two depressed levers (Kaneko, Tamura, Kawashima, & Suzuki, 2006). If a rat released the lever on the incorrect response side during a trial (i.e., error response), then the same tactile stimulation was repeated in the following trial, and the forepaw pressing the correct- or incorrect-response-side lever was automatically elevated by a mechanical device at a preset time after the tactile stimulation (lever activation). This elevation mimics the lever release movement of a voluntary response, which we refer to as a forced movement. Because the timing of lever activations can be preset, we compared the performance improvement rate among different preset times (120, 220, 320, and 420 ms after the tactile stimulation), and examined the timing effects of lever activations on the performance improvement rate of sensory–motor associative learning, as indicated by reductions in ER and RT. As we showed in a previous study (Sano et al., 2013), the performance improvement rate was generally increased by mechanically controlled lever activation on the incorrect response side, but not on the correct response side. Furthermore, we found that lever activations affected performance improvement in a time-dependent manner. In addition, we discuss the most effective timing for the facilitation of performance improvement through motor sensation.

## Method

### Subjects

Thirty-six naïve male Wistar rats (CLEA Japan, Inc., Japan) were used for the experiments. Their weights ranged from 275 to 396 g after the pretraining period, in which the experimenter motivated the rats to perform the task with a reinforcement schedule (Kaneko et al., 2006). The rats were individually housed in home cages in a room maintained at 24 °C with illumination from 7:00 to 19:00. They were deprived of water for 23.5 hours (Brewer, Langel, & Robinson, 2005), while food was freely available. Daily training was conducted for approximately 15 min between 13:00 and 17:00. The rats were rewarded with approximately 10 mL (total) of 3% (w/v) sucrose solution during the task. Additional water was available to the rats for 30 min (19:00–19:30) in their home cages. The rats showed no obvious signs of distress (such as poor grooming, hyper- or hypo-activity, or aggressive behavior). The rats were weighed daily before training to ensure that they maintained more than 85% of the average body weight for animals of the same strain and age fed and provided water ad libitum (see the breeder’s homepage: www.clea-japan.com/en/animals/animal_e/e_03.html, CLEA Japan, Inc., Japan). This water intake regimen allowed the rats to maintain a sufficient level of motivation for the task (Brewer et al., 2005; Mulder, Shibata, Trullier, & Wiener, 2005). All experimental procedures were based on the guidelines of the National Institutes of Health of the United States (1996) and the Japan Neuroscience Society. The experimental protocols for this study were approved by the institutional committee for animal experiments at the National Institute of Advanced Industrial Science and Technology.

### Apparatus

**Fig. 1:**
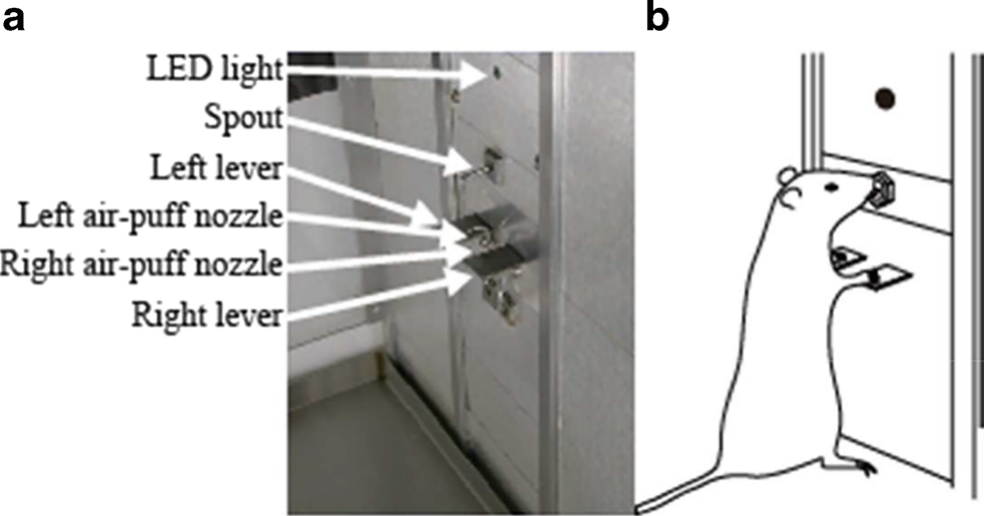
Apparatus. (A) The front panel of the operant box. (B) The rat’s posture in front of the panel during task execution

**Fig. 2:**
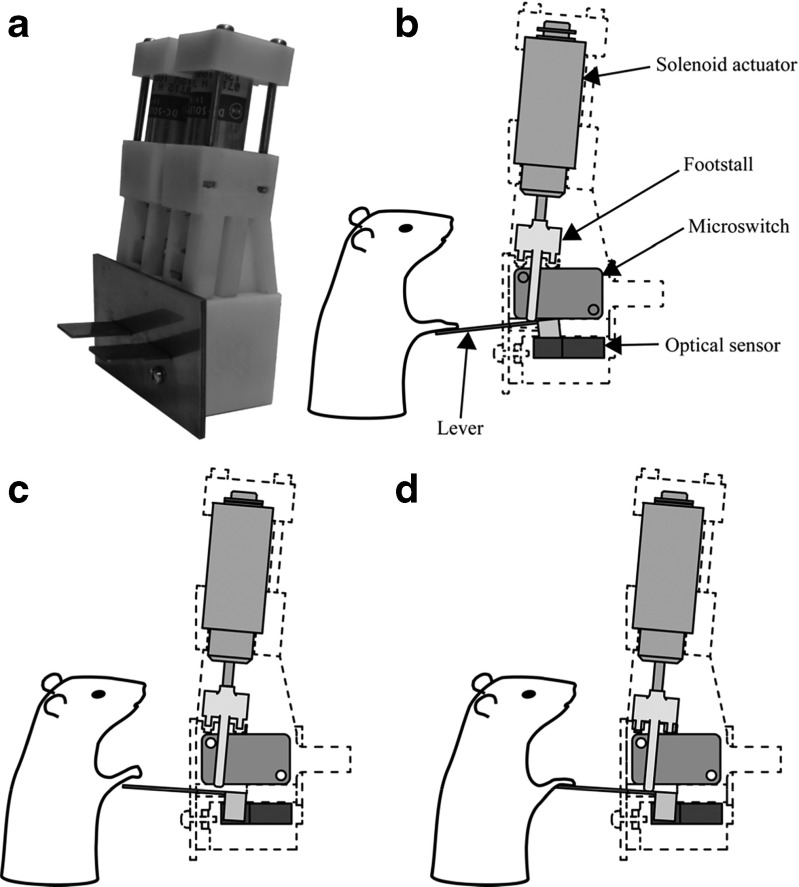
Lever activation device. (A) Photograph of the device with two solenoid actuators placed behind the front panel. (B) Side view of the device and a rat’s posture during the foreperiod, in which both the levers are depressed by the rat. The lever activation mechanism consists of a solenoid actuator, a footstall, a microswitch, and an optical sensor. The footstall moves the lever arm behind the front panel downward by activating the solenoid actuator. The microswitch returns the lever back to the “up” (OFF) position. The lever movements are detected by the optical sensor. (C) During a planned trial, the solenoids and footstalls are not activated, and the rat voluntarily releases one of the levers. Note the gap between the footstall and the lever. (D) During an additional trial, the solenoid actuator moves the footstall downward, and thereby elevates the lever and the rat’s arm

The apparatus was similar to that described in our previous study (Kaneko et al., 2006; Sano et al., 2013). Figure 1A shows the front panel of the operant chamber (30 [L] × 24 [W] × 33 cm [H]; Med Associates Inc., USA), which was placed in a sound-attenuating box (Neuroscience, Japan). Two levers, two air-puff nozzles, one light-emitting diode (LED), and one spout were attached to the operant chamber. The levers, which were 15 mm wide, protruded 20 mm from the front panel and were located 17 cm above the floor, and spaced 13 mm apart. Each lever had an optical sensor (EE-SX670, Omron, Japan) that was set (ON) and reset (OFF) by pressing and releasing the lever, respectively. A lever activation device was placed behind the front panel, as is shown in Fig. 2. The lever activation device had two solenoid actuators (S-75 push-type; Shindengen, Japan), with each actuator depressing the other (hidden) side of the corresponding lever through the footstall. Thus, a depressed lever could move upward by activating the corresponding solenoid actuator. When the actuator was inactive, the lever movements were unhindered because the lever was detached from the footstall. An air-puff nozzle (2.1-mm outer diameter, 1.5-mm caliber) was located 4 mm above its corresponding lever. Each air-puff nozzle was connected to the pressure source (rating pressure of 0.02 MPa) through an electrically controlled valve (UMB1-T1, CKD, Japan). By controlling the valve, a fixed amount of air was delivered to the back of the rat’s forepaw positioned on the corresponding lever. The LED was located 24 cm above the floor, between the levers. The spout protruded 20 mm from the front panel and was positioned 20 cm above the floor, between the levers. Drops of a sugar solution were delivered from the spout (2.1-mm outer diameter and 1.5-mm caliber), which was connected to the container of sugar solution (fluid gauge pressure at the spout: 0.01 MPa) through an electrically controlled valve (UMB1-T1, CKD, Japan). Two beepers were placed behind the front panel; one delivered a sound that indicated that a trial was successfully completed (a train of 2.8-kHz tone bursts at a rate of 700 bursts per minute; M2BJ-BH24E-D, Omron, Japan), whereas the other presented a sound that indicated an error (a 2-kHz continuous tone; M2BJ-B24-D, Omron, Japan). These sounds have been shown to be heard by rats (Guo, Intskirveli, Blake, & Metherate, 2013; Kelly & Masterton, 1977; Sally & Kelly, 1988; Tsytsarev & Tanaka, 2002). A house light and a speaker supplying white noise were fixed in the sound-attenuating box. The sound pressure level at the center of the operant chamber was 57 dB_A_ for white noise, measured using a Digital Sound Level Meter, Type 93411 (CH. BEHA GmbH, Germany). The level increased to approximately 66 dB_A_ when either of the two beepers was activated. A video camera monitored the rat’s behavior. The entire task and reward system was controlled by a custom PC program.

### Procedure

#### Experimental and control groups

A total of 36 rats were assigned to one of the following groups: a control group without lever activations (Control group, *n* = 4); four experimental groups with correct-side lever activations initiated at preset times of 120 ms (C120 group, *n* = 4), 220 ms (C220 group, *n* = 4), 320 ms (C320 group, *n* = 4), or 420 ms (C420 group, *n* = 4) after the air-puff stimulation; and four experimental groups with incorrect-side lever activations initiated at 120 ms (I120 group, *n* = 4), 220 ms (I220 group, *n* = 4), 320 ms (I320 group, *n* = 4), or 420 ms (I420 group, *n* = 4) after the air-puff stimulation. Because the experimental conditions of the normal rats in a previous study by our group (Sano et al., 2013) had been identical to those of the Control, C220, and I220 groups in this article, the data from that article were reanalyzed here for the Control, C220, and I220 groups.

#### Behavioral task

**Fig. 3:**
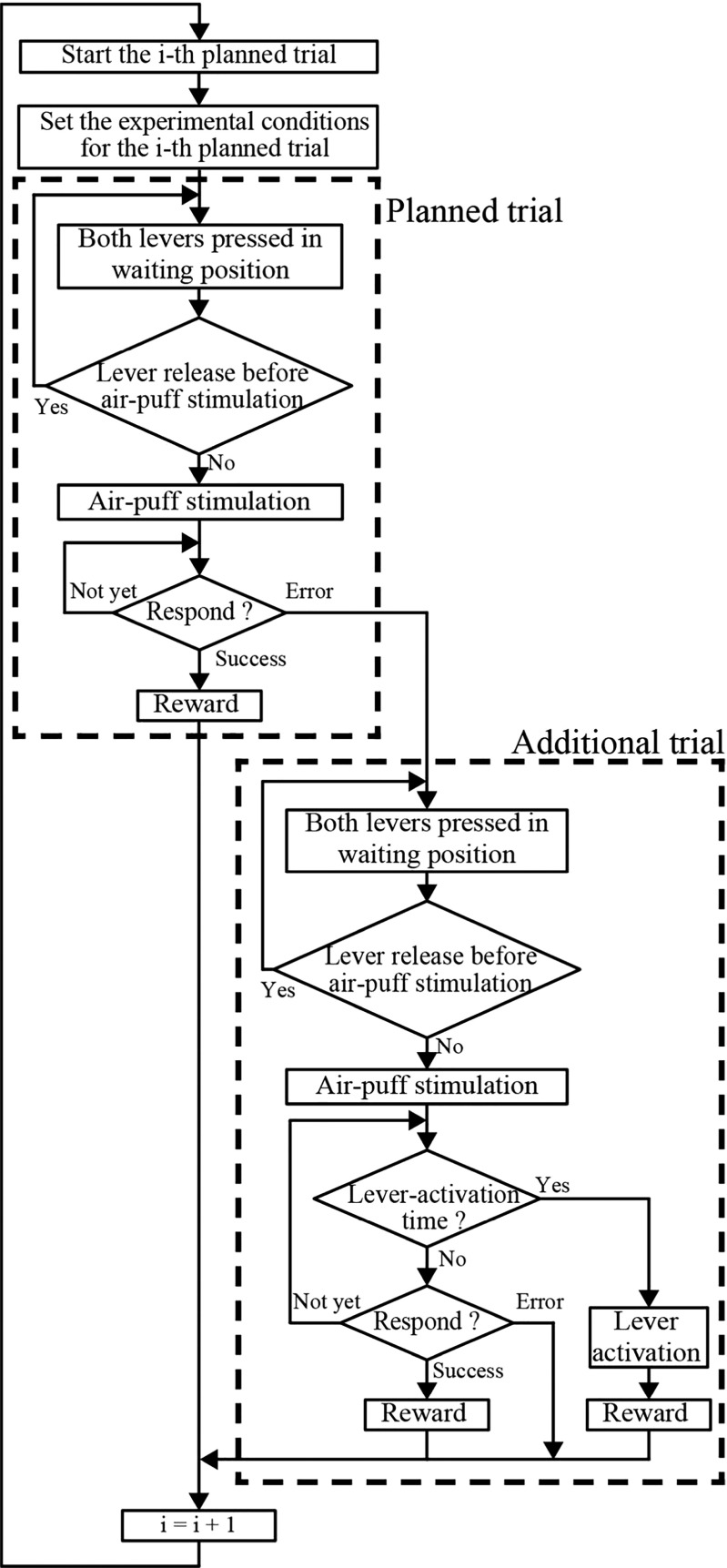
Flow chart. The task consisted of planned and additional trials. An additional trial occurred after every planned trial in which the rat made an error

A choice RT task designed to investigate the effects of spatial stimulus–response compatibility in rats (i.e., the responses to ipsilateral stimuli were faster and more accurate than responses to contralateral stimuli) was used to examine the effects of lever activations on reversal learning (Kaneko et al., 2006; Sano et al., 2013). The rats performed the task in a standing position with their forepaws placed on the two levers (Fig. 1B). The rats were trained to respond to an air-puff stimulus on a forepaw by releasing the lever pressed with the stimulated forepaw (compatible condition) or by releasing the lever pressed with the nonstimulated forepaw (incompatible condition). Figure 3 shows a flowchart that illustrates the task control. A trial began with the rat pressing the two levers with its forepaws (self-pacing). After the rat had depressed both levers, the LED was illuminated to indicate the beginning of a foreperiod (500–1,500 ms). If the rat released a lever in the foreperiod before receiving an air-puff stimulus, the LED was turned off, and the rat repeated the same trial. At the end of the foreperiod, an air-puff stimulus was applied to the back of the forepaw on the left or the right lever. The rat responded to the air-puff stimulus by releasing a forepaw from the depressed lever. The RT was defined as the latency from the onset of the air puff to the release of the left or right lever. In the compatible condition, releasing the lever on the same side where the air-puff stimulus was applied was scored as a correct response, whereas releasing the other lever was scored as an error. The correct and incorrect response sides were switched in the incompatible condition. After a rat had made a response, the LED was turned off. Depending on the response, a sound indicating a correct response or an error was delivered for 1 s immediately after the lever was released. Correct responses were immediately followed by the delivery of a sucrose solution for 200–400 ms (400 ms for RT < 200 ms, 200 ms for RT ≥ 400 ms, with the period being reduced linearly for 200 ms ≤ RT < 400 ms; this time difference in delivery resulted in a difference in the amount of solution delivered, from 0.04 to 0.10 mL). A procedure that varied the reward size RT-dependently was used to encourage the rat to respond to the air-puff stimulus as quickly as possible. An intertrial interval began at the end of the sound indicating a correct or an error response. During the intertrial interval, at least one of the levers should have been in the OFF position. Because any imposed intertrial interval did not exist, the duration of the intertrial interval varied and was always more than 0 ms. The next trial was started by depressing both levers.

We randomly changed the side delivering air-puff stimulation in a sequence of planned trials, and also randomly and uniformly changed the foreperiod duration to 500, 750, 1,000, 1,250, or 1,500 ms. Each combination of stimulus sides and foreperiod durations occurred ten times in a series of 100 trials (Possamaï & Reynard, 1974). Daily training sessions lasted 10–15 min for each rat. If rats had not pressed any lever for 1 min after 10 min of the session, training was terminated. Within this training session, rats were allowed to perform up to 300 planned trials. However, if the rats made an incorrect response in a planned trial, an additional trial was conducted under the same condition. In such additional trials, the stimulus side and foreperiod duration were the same as in the previous error trial. Although we inserted such additional trials into a sequence of planned trials, only rats in the experimental groups received lever activations. The forepaw on the correct response side was elevated in the correct-side lever activation groups (C120, C220, C320, and C420), whereas the forepaw on the incorrect response side was elevated in the incorrect-side lever activation groups (I120, I220, I320, and I420). Because the additional trials, as well as the planned trials, started with the rat depressing both levers with the forepaws, the rat would leave its forepaws on the levers until making a response. Consequently, if the lever activation occurred before the rat had made a response, the solenoid actuator on the lever activation side elevated the forepaw. Unless the rat made an incorrect lever release response before lever activation, a sucrose solution was delivered for 200 ms (constant) as a reward.

#### Reversal learning

**Fig. 4:**
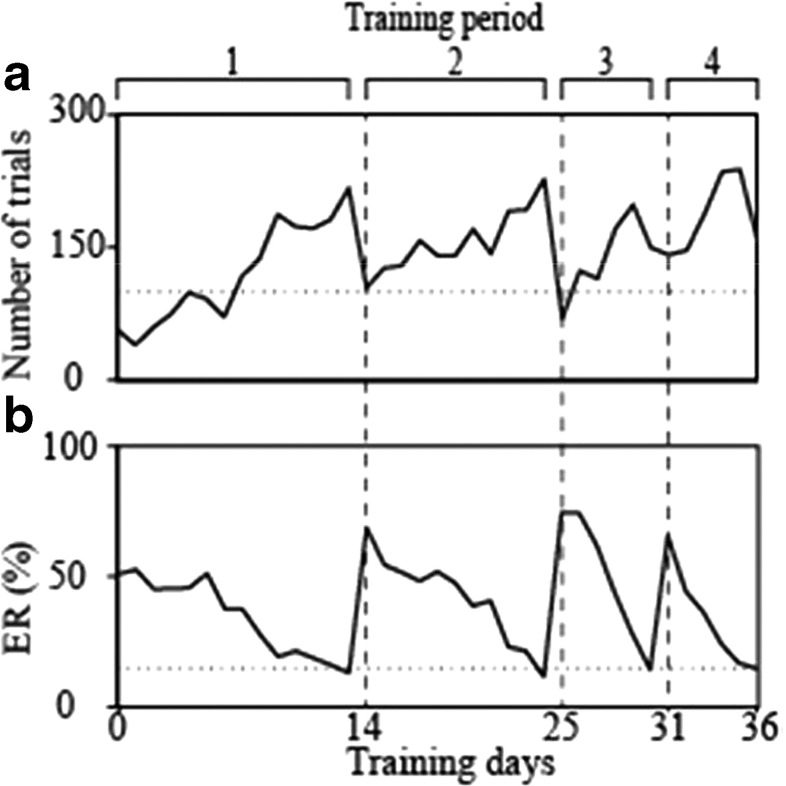
Serial reversal learning. Changes are shown in the number of planned trials (A) and error rates (ERs) (B) over the four training periods. The compatibility condition was reversed between the training periods (three times in total). The dashed lines are boundaries between the training periods. The dotted line in panel A indicates the number of 100 planned trials required for reaching the learning criterion. The dotted line in panel B indicates the ER of 15%, which should be crossed as a rat reaches the learning criterion

After we had trained the rats in all groups, four training periods with three reversals of the compatibility condition (compatible and incompatible) were performed. Half of the rats in each group started the pretraining in the compatible condition, and the other half started the pretraining in the incompatible condition. The compatibility condition was not changed between the pretraining period and the first training period. Each training period consisted of training days in the same compatibility condition, which lasted until the task performance reached the learning criterion of ER < 15% for at least 100 planned trials. Figure 4 shows sample learning data from a rat in the Control group that started with the compatible condition. In each training period, the number of planned trials tended to increase with training days (Fig. 4A), and the ER decreased with training days after the change (reversal) of the correct response side (Fig. 4B). If the rats reached the learning criterion, the compatibility condition of the task was reversed individually.

### Statistical analyses

To detect differences in performance improvement rates among the groups in different lever activation conditions, we analyzed ERs and median RTs for the first five days of the third and fourth training periods. In these training periods, the variations in the rats’ performance were relatively stable after experiencing both the compatibility and incompatibility conditions in the first and second training periods. Performance improvement rates can be determined from the number of days required to reach the learning criterion, which depends only on the ER. However, because the ER change is related to the RT change (Welford, 1980)—for example, through a speed–accuracy trade-off—the performance improvement rate cannot be evaluated solely from the number of training days. Therefore, we analyzed both ERs and median RTs to evaluate performance improvement rates. On the basis of the data from each day, ERs were calculated from planned trials with RT ≤ 1 s, and median RTs were calculated from successful planned trials with RT ≤ 1 s. Trials with RT > 1 s (5.1% of the planned trials) were excluded from the analyses of ERs and median RTs, because ERs reverted to 50% for longer RTs (these data are not shown); therefore, these responses were no longer considered to have been induced by the air puff. We verified that this exclusion had very little effect on our results and conclusions. Because rats reached the learning criterion within four days after reversal, the missing data on Days 4 and 5 were filled in according to the last observation carried forward (LOCF) strategy. The ERs and median RTs were analyzed using a multivariate general linear model, which functioned as a multivariate analysis of variance (MANOVA) with the factors Group (Control, C120, C220, C320, C420, I120, I220, I320, and I420), Day After Reversal (Day 4 or 5), and Training Period (third or fourth). For post-hoc multiple comparisons among the groups, we used Hotelling’s *T* ^2^ test customized for heteroscedastic data, and controlled the false discovery rate by using the Benjamini–Hochberg correction (Benjamini & Hochberg, 1995). To clarify the effects of lever activation side, lever activation timing, and lever activation ratio (i.e., the number of lever activations out of the total number of additional trials), we conducted a statistical analysis using another multivariate general linear model with the factors Lever Activation Side (correct response side or the other side), Lever Activation Timing (120, 220, 320, or 420 ms after air-puff stimulation), Day After Reversal (Day 4 or 5), and Training Period (third or fourth), and with a covariate of lever activation ratio. For the analyses of these general linear models, the Box–Cox transformation was used to eliminate the problems associated with multivariate heteroscedasticity. The uniformity of the groups was confirmed for the data on Day 0 using a multivariate general linear model, similar to a MANOVA, with the factors Group (Control, C120, C220, C320, C420, I120, I220, I320, and I420) and Training Period (third or fourth). The homoscedasticity of the distribution of ERs and median RTs was verified for each group through Box’s *M* test, and the multivariate normality of the residuals was verified by using Mardia’s multivariate normality test. As necessary, the ERs or median RTs were analyzed with univariate statistical tests. All statistical tests were performed in R version 3.2.2 in the Rstudio integrated development environment (version 0.99.486), using the following packages: “biotools,” “car,” “stats,” “mvnormtest,” “Hotelling,” and “MVN.”

## Results

For all 36 rats, data were successfully collected until the fourth training period. The rats performed a median of 223.5 total (planned and additional) trials per day in the third and fourth training periods. For 35 of the rats, the minimum number of total trials per day was 113. The remaining rat performed only 79 total trials (44 planned trials and 35 additional trials) on the day just after reversal. Thirty-four of the rats reached the learning criterion in the fourth training period, but the other two (one in the C120 group and the other in the C220 group) were retired before reaching the criterion. The missing data for one I420 rat from Days 4 and 5 in the fourth training period were filled in by following the LOCF strategy—that is, the data obtained in Day 3 were replicated as the data for Days 4 and 5. The data from the third and fourth training periods (i.e., the data for different compatibility conditions) were pooled to increase the reliability of the statistical analyses, because the compatibility condition and its interactions with the lever activation condition were not statistically significant (data not shown). The effects of lever activations on performance improvement rates were analyzed using the differences in ERs and RTs.

### Time-to-event analysis of training days

**Fig. 5:**
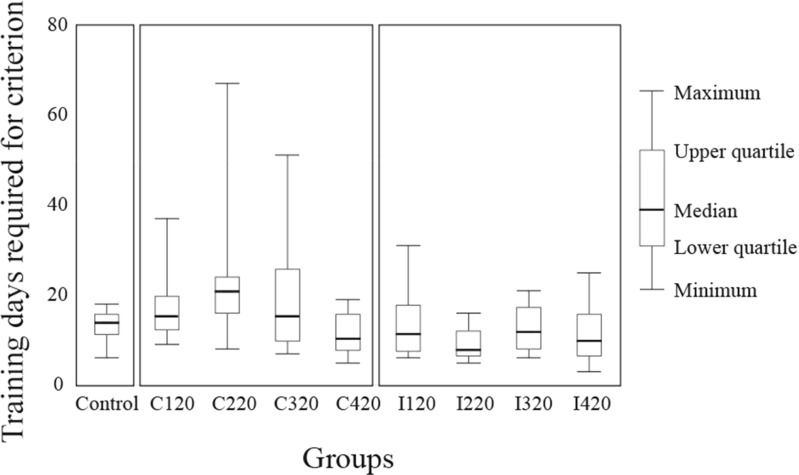
Training days required for task learning. The number of training days required for reaching the learning criterion is indicated. The data were collected from all rats in the third and fourth training periods

The numbers of training days required for the rats to reach the criterion are summarized for each group in Fig. 5. For a fundamental analysis of the data, we conducted a semiparametric time-to-event analysis—that is, a Cox proportional hazard regression analysis with the factors Group (Control, C120, C220, C320, C420, I120, I220, I320, and I420) and Training Period (third or fourth). There was no violation of the proportional hazards assumption. As a result, although the effect of group was significant [χ^2^(8) = 16.5, *p* = .036 < 0.050, likelihood-ratio test for group], we could not detect any significant difference between the groups in post-hoc multiple comparisons. However, for data excluding those of the Control group, we detected a significant effect of lever activation side in a Cox proportional hazard regression analysis with the factors Lever Activation Side, Lever Activation Timing, and Training Period [χ^2^(1) = 6.03, *p* = .014 < .05, likelihood-ratio test for lever activation side]. No significant effects (except for lever activation side) were detected for the effect of training period [χ^2^(1) = 2.40, *p* = .121, likelihood-ratio test for training period] or for the interaction between lever activation side and lever activation timing [χ^2^(3) = 5.63, *p* = .131, likelihood-ratio test for the interaction].

### Effects of training days after reversal

**Fig. 6:**
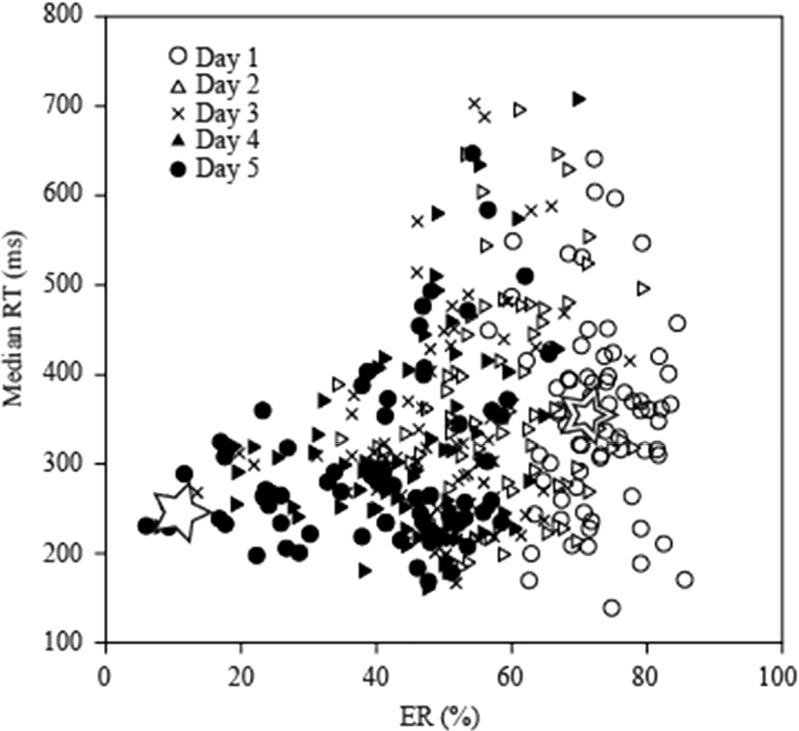
Distribution of daily error rates (ERs) and median reaction times (RTs). The distribution of the data five days after reversal in the third and fourth training periods indicate that the relationship between ERs and median RTs differed from day to day. The open star and the double open star indicate the means of the ERs and RTs on Days 0 and 1, respectively

**Fig. 7:**
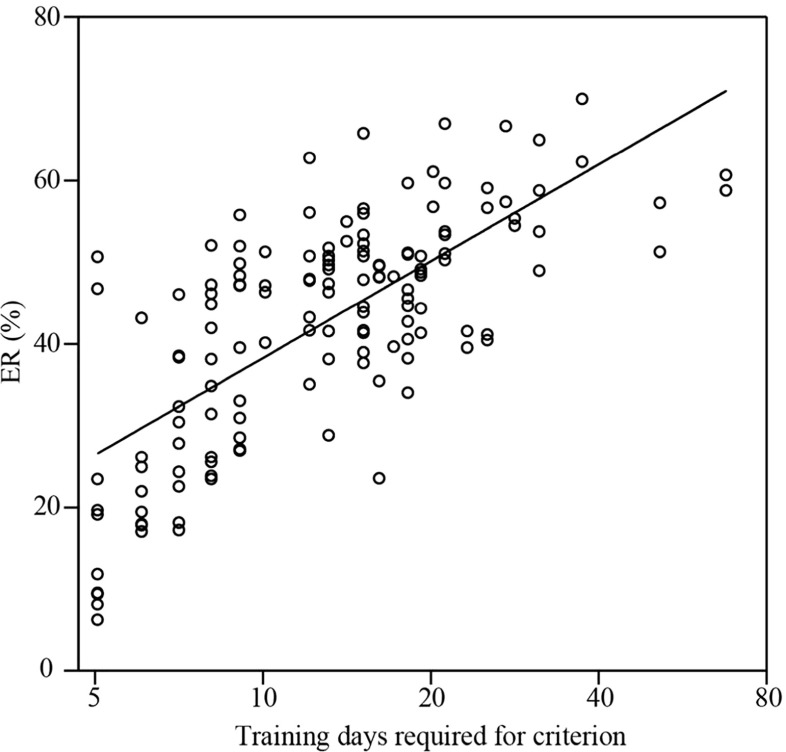
Correlations between the number of training days required for reaching the learning criterion and the ERs on Days 4 and 5 after reversal. The data were collected from all rats in the third and fourth training periods. The graph indicates a relationship between ER and the number of training days required for reaching the learning criterion. The solid line is a fitted logarithmic curve. The approximate formula is *y* = ln(*x*) – 1.14 (*y*, ER; ln, natural-logarithm function; *x*, number of days for reaching the criterion). The coefficient of determination (*R* ^2^) was .501. The horizontal axis is logarithmic

Progressive changes in the relationship between ERs and RTs for all rats during the first five days after reversal are shown in Fig. 6. For reference, we indicate the mean of ERs and median RTs on Day 0 (before reversal) with an open star in the figure, and those on Day 1 (immediately after reversal) with a double open star. Because the mean values of ERs and median RTs on Day 0 are regarded as the learning goal, the mean center for ERs and median RTs moved from the double open star to the open star over the course of time. Thus, both ERs and median RTs tended to decrease with performance improvement. It should be noted that, during the first five days after reversal, the RTs were concentrated in the range 200–300 ms (mean 346 ms, median 294 ms, 210-ms center of the most frequent bin among 20-ms bins), indicating that lever activation timings were within the range of RTs. The difference in ERs and median RTs between two successive days was significant (statistical results are not shown) but diminished gradually after reversal. On Days 4 and 5 after reversal, the effect of training days was no longer significant [*F*(2, 132) = 2.72, *p* = .070, MANOVA with the factors Group, Day After Reversal, and Training Period]. At this level, whereas the median RTs were weakly correlated with the number of training days required to reach the learning criterion (*R* ^2^ = .083), the ERs were highly correlated (*R* ^2^ = .501; see Fig. 7). Thus, the performance on Days 4 and 5 reflected the performance improvement rate of reversal learning in the present task.

### Differences in ER and RT between the groups

**Fig. 8:**
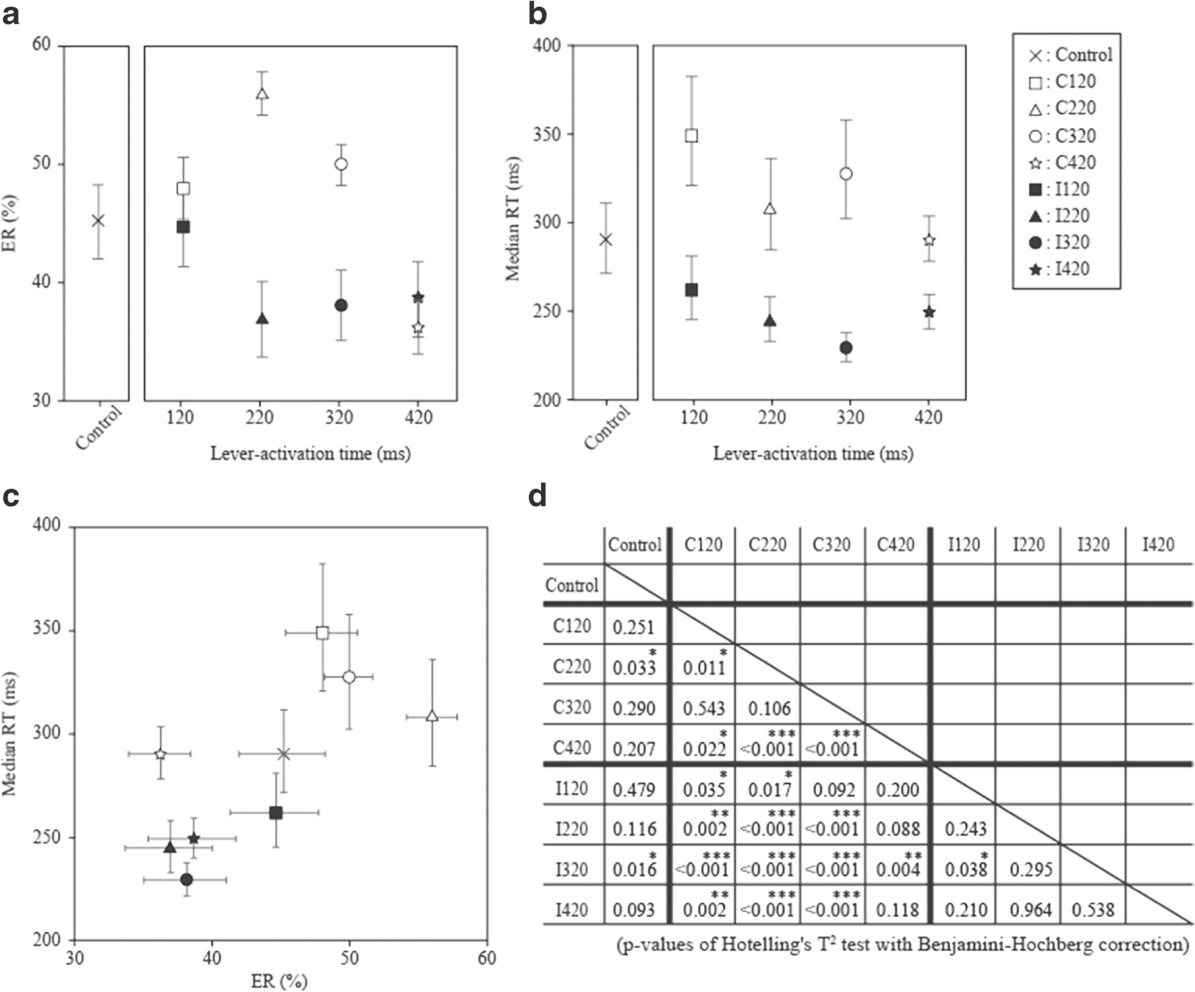
Effects of lever activation condition on ERs and median RTs. Data collected on Days 4 and 5 after reversal were analyzed. The mean values of the ERs and median RTs for the different lever activation groups are shown in panels A and B, respectively. Their distribution in the *x*–*y* plane (*x*, ER; *y*, median RT) is shown in panel C. Post-hoc multiple comparisons were conducted by using Hotelling’s *T* ^2^ test with control of the false discovery rate using the Benjamini–Hochberg correction. The *p* values for multiple comparisons between groups in the rows and columns are indicated in panel D. Incorrect-side lever activation groups tended to have lower ERs and shorter median RTs than the other lever activation groups. These tendencies were more apparent for groups in which lever activations were applied to rats that made more responses—that is, the C220 and I320 groups. Error bars denote *SEM*s, and asterisks indicate significance: ^*^ *p* < .050, ^**^ *p* < .010, ^***^ *p* < .001

The ERs and median RTs on Days 4 and 5, which reflect the performance improvement rate of reversal learning, differed between groups [*F*(16, 266) = 5.29, *p* < .001, MANOVA with the factors Group, Day After Reversal, and Training Period]. Figure 8 shows the mean values of ERs and RTs on Days 4 and 5 for the groups, as well as the results of statistical comparisons. As is shown in Fig. 8C, the mean center of ERs and RTs for the Control group—that is, the no-lever-activation group—was surrounded by those for the other groups. Although one exception existed (i.e., C420 group), the correct-side lever activation groups exhibited relatively higher ERs and longer RTs than the Control group. By contrast, the incorrect-side lever activation groups had lower ERs and shorter RTs. This tendency was confirmed by post-hoc multiple comparisons using Hotelling’s *T* ^2^ test with the Benjamini–Hochberg correction (shown in Fig. 8D).

### Effects of lever activation conditions

The groups other than the Control group can be seen as combinatorial combinations of two lever activation sides (correct and incorrect response sides) and four lever activation timings (120, 220, 320, and 420 ms after the air-puff stimulation). By constructing a multivariate general linear model for the data from these groups, we conducted a detailed statistical analysis of lever activation conditions—that is, lever activation side and timing. Because the slower lever activation timing groups (e.g., C420 or I420 group) had fewer lever activations by rats making voluntary responses prior to lever activation, the lever activation ratio can be seen as a factor related to lever activation timing. However, it is not fully dependent on lever activation timing, because the number of lever activations also depends on the number of error occurrences, which is an uncontrollable factor. Therefore, we included the lever activation ratio as a covariate in the model and evaluated the effects of lever activation conditions by considering the effects of the lever activation ratio.

We constructed an initial multivariate general linear model with the factors Lever Activation Side, Lever Activation Timing, Day After Reversal, and Training Period, and with the covariate lever activation ratio; we also included the interactions between lever activation side and lever activation timing and those between lever activation side and lever activation ratio. In the stepwise reduction procedures of the initial model using Akaike’s information criterion (AIC), the lever activation ratio covariate and its interaction disappeared, indicating that they did not remain significant factors. The statistical results of the final model after the stepwise reduction procedures are summarized in Table 1. The data indicate that both lever activation side and timing were significant as main factors, and that their interaction was also significant. The results of the post-hoc multiple comparison are not shown here, but the detailed results are shown in Fig. 8D: Highly significant differences were observed between the C220 and I220 groups and between the C320 and I320 groups, and less significant or insignificant differences were observed between the C120 and I120 groups and the C420 and I420 groups.

**Table 1:** Results of a detailed analysis using a multivariate general linear model

Factors	Pillai’s Trace	*F* Value	*df*1	*df*2	*p*
Lever Activation Side	.334	29.3	2	117	<.001^***^
Lever Activation Timing	.192	4.19	6	236	<.001^***^
Day After Reversal	.041	2.52	2	117	.085
Training Period	.049	3.00	2	117	.054
Interaction Between Lever Activation Side and Timing	.151	3.21	6	236	.005^**^

Four factors (i.e., Lever Activation Side, Lever Activation Timing, Day After Reversal, and Training Period), one covariate (lever activation ratio), and two interactions (between lever activation side and lever activation timing, and between lever activation side and lever activation ratio) were included in the initial multivariate general linear model. The initial model was simplified using stepwise procedures based on Akaike’s information criterion. Over the course of these stepwise reduction procedures, the covariate of the lever activation ratio and its interaction disappeared. The final model was used for statistical analysis, and its results are shown as a MANOVA report. Asterisks indicate significance: ^**^ *p* < .010, ^***^ *p* < .001

### Forepaw releases opposite the lever activation side

**Fig. 9:**
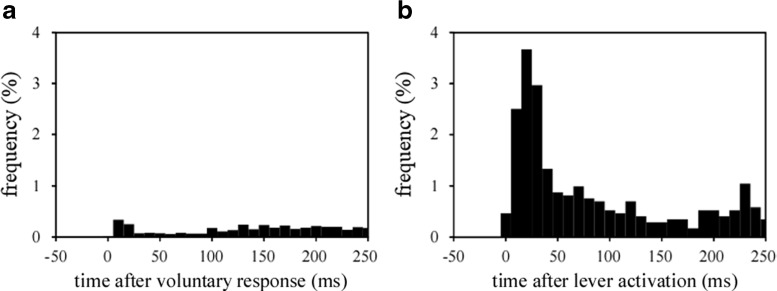
Histograms of lever releases. (A) Lever releases of the non-responded-side forepaw after voluntary correct responses on Day 0. (B) Lever releases of the forepaw opposite the lever activation side in additional trials with lever activations (i.e., in lever activation trials) on Day 1. Rats ordinarily released a lever as a voluntary response. However, in lever activation trials, not only the lever-activation-side forepaw, but also the other forepaw was often elevated. This effect is demonstrated by a peak approximately 25 ms after the lever activation in panel B—that is, rats released the nonactivated lever approximately 25 ms after lever activation. The bin width is 10 ms

For more detailed consideration, histograms of the lever releases of the forepaw opposite the correct response side or lever activation side of the rats in the experimental groups (weights on Days 0 and 1 in the third and fourth training periods = 323–581 g) are shown in Fig. 9. The rats ordinarily released a lever as a voluntary response. During this period, the other forepaw was still depressing the lever (Fig. 9A). However, in additional trials with lever activation (i.e., lever activation trials), the lever-activation-side forepaw was elevated, and subsequently the other forepaw occasionally released the lever. Figure 9B shows a peak approximately 25 ms after lever activation—that is, the rats released the nonactivated lever approximately 25 ms after lever activation. Similar lever releases of the forepaw opposite the lever activation side were observed even after the rats had reached the learning criterion, and the most frequent bin of the histogram was the same as in Fig. 9B. Therefore, we assume that the lever releases of the forepaw opposite the lever activation side occurred consistently with a certain frequency (11.4%; the value was calculated by accumulating the five bins from 5 to 55 ms [corresponding to the primary peak] after lever activation; Fig. 9B) due to the mechanical reactions of rats’ bodies to lever activations. Because the rats conducted the task in a standing position, as is shown in Fig. 1B, and because a forepaw was occasionally moved upward immediately after the other forepaw’s elevation by the lever actuator (Fig. 9B), the forepaw opposite the lever activation side was presumed to have been moved upward by the rat’s body swinging back after the lever activations.

## Discussion

We investigated the effects of lever activations on learning a sensory–motor association task in rats. We successfully demonstrated facilitation and hindering of performance improvement as a function of the side and timing of lever activations. Here we discuss possible reasons why the performance improvement rate was altered by lever activations. We postulate that the congruence and timing of the motor sensation artificially provided by lever activations were important factors in facilitating the learning process.

### Evaluating performance improvement rate using ER and RT

We showed that lever activation substantially affected the number of training days required to reach the learning criterion after reversal (see the Time-to-Event Analysis of Training Days subsection). Weak effects of lever activation were detected for two factors in the semiparametric statistical tests—that is, Group (*p* < .05) and Lever Activation Side (*p* < .05). However, it should be noted that the effects of lever activation gradually decreased after reversal, because the reduction in ERs with training days decreased the number of lever activations. Therefore, lever activation must have had a larger effect at the beginning of the training period after reversal. Accordingly, we analyzed the data from the early training period (first five days after reversal) to determine the effects of lever activation on performance improvement rates.

Furthermore, we analyzed the data with attention to RTs as well as ERs, because the effects of performance improvement should appear in both ERs and RTs. We found that the ERs on Days 4 and 5 after reversal were related to the number of training days required to reach the learning criterion after reversal (see the Effects of Training Days After Reversal subsection). Thus, it is reasonable to use the ER to evaluate the performance improvement rate. However, using only the ER would lead us to incorrectly evaluate performance improvement rates, because RTs can also reflect the performance improvement rate. This occurs not only because the ER change is related to the RT change (Welford, 1980)—for example, the speed–accuracy trade-off—but also because RT decreases as task performance progresses. For example, the RT decreases after the ER improves less than ~50% (Brooks, Hilperath, Brooks, Ross, & Freund, 1995; Brooks, Kennedy, & Ross, 1983). Indeed, longer RTs were observed when ERs were higher than ~50% (Fig. 6), but not in the range of lower ERs. Furthermore, a reduction of reward with longer RTs made the rats respond as quickly as possible. Therefore, even if the ERs did not differ between groups, performance improvement would be predicted to be faster in groups with shorter RTs than in those with longer RTs. Because we needed to evaluate both ERs and median RTs to compare performance improvement rates, we used a multivariate general linear model for our statistical analyses.

### Effects of lever activation side on performance improvement rate

Although no statistically significant differences in ERs and RTs were detected between the experimental and control groups at the beginning of reversal learning, significant differences between the groups appeared on Days 4 and 5 after reversal (Fig. 8D), indicating that performance improvement rates were altered by the lever activation conditions. As is summarized in Table 1, Lever Activation Side was the main factor affecting the learning process. Because both ERs and median RTs decreased as task performance improved, the lower ERs and shorter median RTs for the incorrect-side lever activation groups in Fig. 8C indicate the facilitative effects of incorrect-side lever activation. This facilitative effect was apparent in the I320 group, as is shown in Fig. 8D; in particular, the I320 group had the shortest mean value of median RTs among the groups (Fig. 8B). By contrast, the C220 group had the highest mean ER value among the groups, suggesting a hindering effect on performance improvement (Fig. 8A). Thus, these results indicate that both facilitative (I320 group) and hindering (C220 group) effects of lever activations operate on performance improvements, and that the side of lever activation is an important factor that determines whether the effects will be facilitative or hindering.

### Effects of lever activation timing on performance improvement rate

Incorrect-side lever activation groups exhibited faster performance improvement, whereas correct-side lever activation groups exhibited slower performance improvement. In addition, lever activation timing exerted larger effects near the response timing for both the correct- and incorrect-side lever activations—that is, ER was higher when the correct-side lever activation was applied near the response timing (see open symbols in Fig. 8A), and RT was shorter when the incorrect-side lever activation was applied near the response timing (see closed symbols in Fig. 8B). Table 1 shows the effect of lever activation timing and its interaction with lever activation side.

These timing effects cannot be fully attributed to the relationship between ER and RT. For example, ER and RT exhibit a speed–accuracy trade-off (Welford, 1980)—that is, ER can increase as RT decreases. Therefore, the highest ER in the C220 group could result from the shortest RT in the correct-side lever activation groups. However, the median RT did not differ significantly between the correct-side lever activation groups (Fig. 8B; this was confirmed by univariate statistical comparisons, but the results are not shown). Therefore, the effects of lever activation timing on ER cannot simply be explained in terms of a speed–accuracy trade-off. Furthermore, the statistical insignificance of the differences in RT among the correct-side lever activation groups means that no relationship between ER and RT would have caused the timing effects of lever activation on performance improvement rate. This was also the case for the incorrect-side lever activation groups (i.e., the differences in ERs among the groups were statistically insignificant; see Fig. 8A; this was also confirmed by univariate statistical comparisons, but the results are not shown).

Likewise, the number of lever activations was not a cause of the timing effects of lever activation. If the number of lever activations affected performance improvement rate, the extent of facilitation or hindrance would be highest in the 120-ms groups and lowest in the 420-ms groups, because the number of lever activations decreased with lever activation time. However, the facilitative and hindering effects of lever activations were remarkable in the I320 and C220 groups, respectively, but not in the I120 and C120 groups (Fig. 8). Thus, the timing effects of lever activation on performance improvement rate cannot be explained by the number of lever activations. This is also demonstrated by the fact that the lever activation ratio disappeared from the factors used in the final reduced general linear model.

Consequently, to explain the results, we propose that lever activation affected performance improvement rate in a time-dependent manner, and that those effects were apparent when lever activation timing was near the rats’ reaction times—that is, 200–300 ms after the air-puff stimulation.

### Contribution of motor sensation to performance improvement

The difference in performance improvement rates between the groups resulted from motor sensation. The planned trials and the additional trials with responses before lever activation should have been conducted in the same way among the groups. A difference between groups was only observed after lever activation: Either motor sensation was induced by correct-response-side lever activations (C120, C220, C320, and C420) or incorrect-response-side lever activations (I120, I220, I320, and I420), or motor sensation was accompanied by voluntary responses (Control). Although motor sensation induced in the lever activation groups should have been similar in the repetitions of lever activations, the motor sensations arising in the Control group were inconsistent on the correct response side (as a success) or the incorrect response side (as an error). Such differences in motor sensation between groups in the additional trials should have affected the learning process. Moreover, factors other than motor sensation (e.g., aversion, auditory inputs, and the visual inputs produced by lever activation) might also have affected performance improvement rates. If lever activations were aversive for rats, the number of trials conducted should be reduced (thus performance improvement rates would decrease [i.e., performance improvement would be hindered] in the correct-side lever activation groups), and RTs would be shorter than the lever activation timings in order to avoid lever activations (thus, incorrect-side lever activation groups with shorter RTs would be considered as facilitating effects). However, the number of total trials (planned and additional trials) did not differ significantly before and after reversal [applying the Box–Cox transformation to the data, *F*(1, 118) = 0.155, *p* = .695, in an ANOVA with the factors Lever Activation Side, Lever Activation Timing, Day (Day 0 or 1), and Training Period; grand means: Day 0 = 216 trials ± 5.69 (*SEM*), Day 1 = 218 trials ± 4.50 (*SEM*)]. Furthermore, although the number of lever activations increased immediately after task reversal, the median RT elongated for all of the lever activation groups [applying the Box–Cox transformation to the data, *F*(1, 118) = 68.2, *p* < .001, in an ANOVA with the factors Lever Activation Side, Lever Activation Timing, Day (Day 0 or 1), and Training Period; grand means: Day 0 = 251 ms ± 6.04 (*SEM*), Day 1 = 362 ms ± 12.9 (*SEM*)]. Therefore, we conclude that lever activations were not strongly aversive stimuli for the rats. If auditory sensation affected performance improvement rates, lever activations accompanied by auditory stimulation—for example, in groups C220 and I220—would have affected performance improvement rates in the same way. However, we observed a hindering effect of lever activation on performance improvement rates in the C220 group, but not in the I220 group. Because these two groups exhibited no differences except for lever activation side, we conclude that auditory sensation did not cause a difference in the performance improvement rates between these two groups. A similar effect was observed in the C320 and I320 groups: I320 exhibited a facilitative effect of lever activation, whereas C320 did not. Thus, the sound sensation induced by lever activation did not cause a significant difference in performance improvement rates between the correct-side and incorrect-side lever activation groups. Similarly, visual sensation did not cause such a difference. This is because the vertically narrow visual field of rats did not allow them to see their forepaws and levers when in the standing position (Hughes, 1979). Thus, the visual sensations induced by lever activation could not have resulted in a difference in performance improvement rates between the correct-side and incorrect-side lever activation groups. Consequently, the primary influence on performance improvement rates must have been the side on which lever activations were induced—that is, differences in the motor sensations accompanied by the lever activations.

### Congruency in motor sensation separates facilitative and hindering effects

**Fig. 10:**
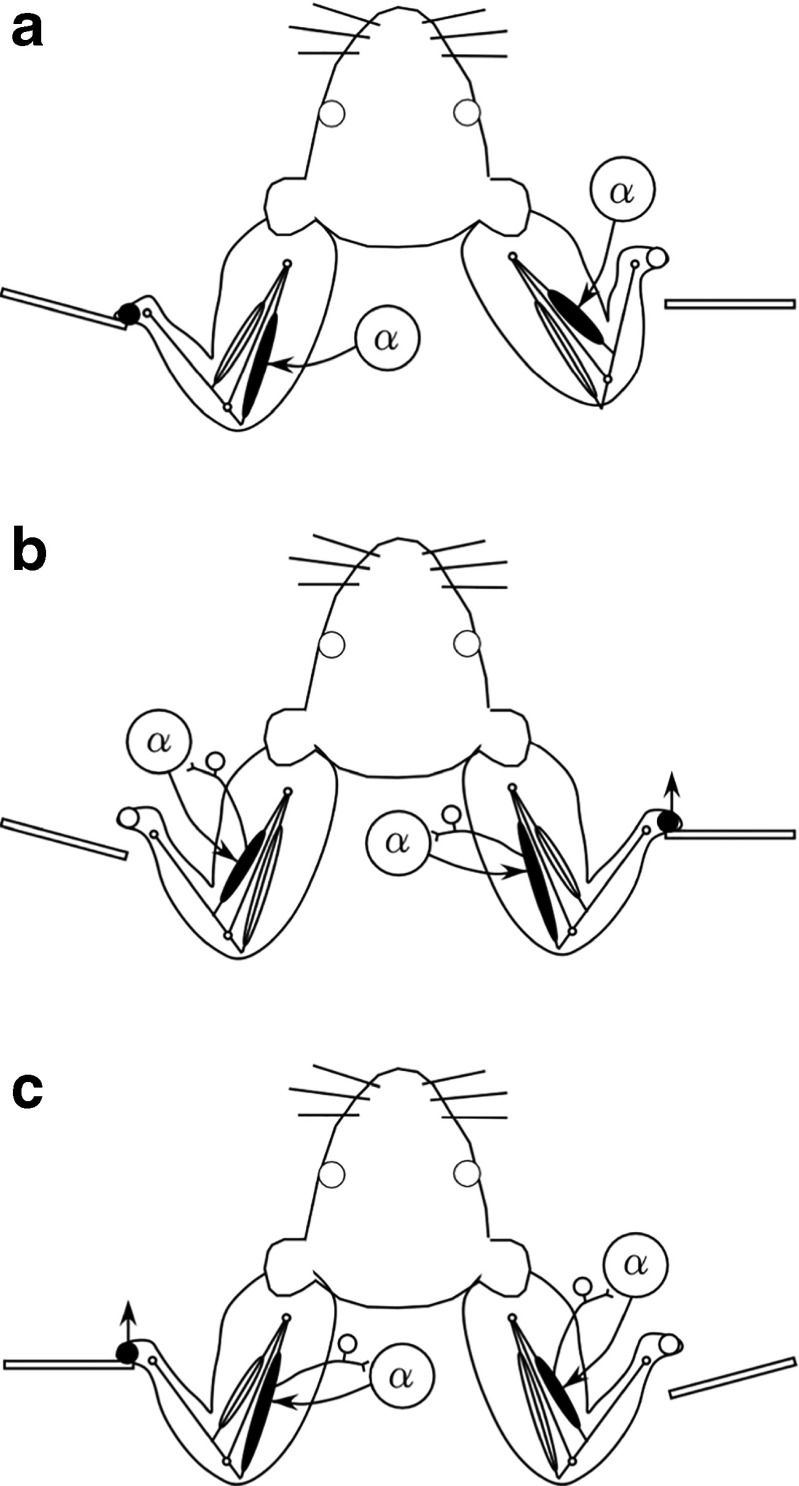
Hypothetical schematic diagrams. In the trials shown in diagrams A–C, the correct response side is assumed to be on the right. (A) Voluntary correct response of the right forepaw. (B) Lever activation on the correct response side (right). (C) Lever activation on the incorrect response side (left). The open and closed circles on forepaws indicate the reduction and increase in lever-press force, respectively. Spinal α motor neurons innervating the brachial biceps and triceps are presumed to be active in voluntary movements and to be activated by the stretch reflex caused by lever activation. The reduction in lever-press force of the forepaw opposite the lever activation side (left in B, and right in C) is considered to be caused by the rat’s body being swung back by lever activation. Together, the three diagrams indicate that lever activation on the incorrect response side (C), but not on the correct response side (B), causes changes in forelimb neuromuscular activities and cutaneous afferent activities similar to those associated with voluntary correct responses (A)

Lever activations had both facilitative and hindering effects on performance improvement. The primary cause underlying these contrary effects may have been the difference between the motor sensations induced by activations of the correct- and incorrect-response-side levers. In a previous study (Sano et al., 2013), we found that lever activations on a forepaw frequently caused lever releases of the other forepaw by inducing body movements—for example, the rat’s body swinging back. Such body movements induced by incorrect-side lever activations were presumed to induce lever releases of the correct-side forepaw and stretch-reflex-like feedback to motor neurons innervating the brachial biceps of the correct-side forelimb. Similarly, in the present experiment, the forepaw opposite the lever activation side was occasionally released from the lever (Fig. 9B), but the frequency of such lever releases was not very high; short-latency releases (5–55 ms after lever activation) occurred in about 11% of the lever activation trials. At the same time, the forepaw on the lever activation side remained on the lever until the lever activation time, and consequently moved upward upon lever activation. This occurred because, if a lever was released before the lever activation time, lever activation was not applied in that trial. Furthermore, in a separate preliminary experiment (details omitted for simplicity), we measured lever-press force during the lever activations and releases using three other rats (Wistar, weight: 654–764 g). These results indicated that, although the rats did not release the forepaw from the lever opposite the lever activation side within 5–55 ms after lever activation in most of the lever activation trials (~98%; the forepaw release rate [~2%] was lower than that in the main experiment [~11%], possibly due to the greater inertia of the body weights of these three rats [654–764 g] relative to those of the rats used for Fig. 9 [323–581 g]), the lever-press force of this forepaw was reduced from the value at lever activation time in a majority (~83%) of lever activation trials. The variation in the reduction of lever-press force may have been responsible for the temporal variation and frequency of lever release opposite the lever activation side (Fig. 9B). We also found that the lever-press force on the lever activation side was positive (lever depression) at the lever activation time for most of the lever activation trials (~97%), indicating that the forepaw on the lever activation side was almost always elevated passively by the lever actuator and remained on the elevated lever. Thus, the stretch-reflex-like feedback was presumed to occur consistently in the forepaw on the lever activation side (stretching brachial triceps), and less frequently in the other forepaw (stretching brachial biceps). On the basis of these observations, we made schematic diagrams related to changes in lever-press force and the activities of motor neurons that is accompanied by voluntary movements and induced by stretch-reflex-like feedback, shown in Fig. 10. A comparison of the schematic diagrams reveals that changes in the lever-press force and activities of motor neurons were similar between voluntary forepaw movements (Fig. 10A) and incorrect-response-side lever activations (Fig. 10C), but not between voluntary forepaw movements (Fig. 10A) and correct-response-side lever activations (Fig. 10B). Such similarity and dissimilarity in motor sensations may be responsible, respectively, for the facilitative and hindering effects on performance improvement. Thus, we propose that the main factor responsible for facilitating the learning process was congruency in the motor sensations between voluntary release of the correct-side lever and forced movement induced by incorrect-side lever activations. Because motor sensation involves several afferent inputs—for example, cutaneous afferent inputs, proprioceptive Ia afferent inputs from muscle spindles, and Ib afferent inputs from Golgi tendon organs—future studies should investigate how these afferent inputs contribute to the facilitation or hindrance of performance improvement.

### Possible cause for the timing effects of motor sensation

In addition to lever activation side, the timing of lever activation also affected performance improvement. Indeed, whereas the mode value for the rats’ RTs was in the range 200–300 ms, the C220 and I320 groups were affected more strongly by lever activation than other groups (Fig. 8). The timings at which lever activation most greatly affected performance improvements were near the RTs, as is demonstrated by the differences between the C220 and I220 groups and between the C320 and I320 groups. This observation indicates that there is an optimal timing at which the application of motor sensation can intervene in the learning processes for a sensory–motor association task, and that this point is near the time when responses occur—that is, during the motor execution phase followed by the response evaluation phase. Thus, motor sensation induced by lever activation was presumed to have intervened in some process or processes relating to the task response, rather than to the task cue stimulus—for example, processes of motor command execution and response evaluation in the central nervous system. With regard to neurophysiological mechanisms, we hypothesize that neural plasticity in the central nervous system can be induced by associating motor intent with artificially generated movement and afferent activity with a certain timing precision (Ethier et al., 2015). Alternatively, the salience of motor sensation artificially induced during the motor execution phase may modulate the response evaluation process required for error correction (Rumbaugh, King, Beran, Washburn, & Gould, 2007; Stock, Wascher, & Beste, 2013). In any case, further studies will be required in order to elucidate the underlying neurophysiological mechanisms.

## Conclusion

We investigated the effects of forced movements on the acquisition of a choice RT task in a two-lever operant conditioning chamber. Forced movements were produced by a mechanical device that activated (elevated) the lever on the correct or incorrect response side between 120 and 420 ms after the presentation of air-puff stimulation. These forced movements changed performance improvement rates, which were slowest in the group with correct-side lever activation at 220 ms, and fastest in the group with incorrect-side lever activation at 320 ms. The results demonstrate that, even in the context of learning a sensory–motor association task that does not require closed-loop control, motor sensation can intervene in the learning process. Our findings indicate that an appropriate timing of motor sensation induced by lever activation is important in facilitating task learning. If the motor sensation is congruent with voluntary response movements near the response timing, then forced movements can facilitate task learning. On the basis of this evidence, we suggest that the congruence and timing of feedback are key to interventions in sensory–motor associative learning through motor sensation. Further studies will be required in order to elucidate the underlying neurophysiological mechanisms.
